# Comprehensive Powder Rheological Characterization of Fifteen Lactose-Based Co-Processed and Single-Component Excipients Using FT4 Powder Rheometry and European Pharmacopoeia Methods: A Multi-Parameter Comparative and Correlative Study

**DOI:** 10.3390/pharmaceutics18050558

**Published:** 2026-04-30

**Authors:** Martin Röttig, Bertram Wolf, Jessica Zwanzig, Fabian Herz, Florian Priese

**Affiliations:** Department of Applied Biosciences and Process Engineering, Anhalt University of Applied Sciences, Bernburger Str. 55, 06366 Köthen, Anhalt, Germany; bertram.wolf@hs-anhalt.de (B.W.); jessica.zwanzig@hs-anhalt.de (J.Z.); fabian.herz@hs-anhalt.de (F.H.); florian.priese@hs-anhalt.de (F.P.)

**Keywords:** co-processed excipients, FT4 powder rheometer, pharmacopoeia, powder flow, lactose, direct compression, shear cell, correlation analysis, particle size distribution

## Abstract

**Background/Objectives**: Co-processed excipients (CPEs) are designed for direct compression through particle engineering, yet comprehensive powder rheological profiles systematically comparing advanced and traditional characterization methods remain limited. This study characterized fifteen lactose-based excipients using European Pharmacopoeia (Ph. Eur.) methods and the complete Freeman FT4 Powder Rheometer measurement suite, establishing a correlation framework linking particle-level attributes to macroscopic flow behavior. **Methods**: Fifteen excipients were characterized for bulk and tapped density, compressibility index, flow time (Ph. Eur. 2.9.16), and angle of repose (Ph. Eur. 2.9.36). Particle size and shape were measured by dynamic image analysis. FT4 measurements comprised stability and variable flow rate testing, consolidation, aeration, compressibility, permeability, shear cell, and wall friction at three surface roughness. Pearson correlation matrices were computed across all 53 parameters. **Results**: Classical flow indices classified most CPE as good-to-satisfactory, failing to discriminate materials with fundamentally different dynamic flow profiles. FT4 testing revealed a fourfold range in Basic Flowability Energy (624–2107 mJ), a ninefold range in flow function coefficient (4.3–35.8), and wide aeration sensitivity differences (Aeration Ratio: 1.9–283.7). Strong correlations were identified between Specific Energy and compressibility index (r = 0.85), cohesion and Flow Rate Index (r = 0.79), and Normalized Aeration Sensitivity and pressure drop (r = 0.86). Within-family comparisons (Tablettose 70/80/100, FlowLac 90/100) revealed that particle size distribution breadth is a more critical flow determinant than median size alone. **Conclusions**: Combining FT4 rheometry with pharmacopoeial testing provides substantially greater discriminating power than either approach alone, enabling rational excipient selection for direct compression formulation.

## 1. Introduction

Solid oral dosage forms remain the predominant pharmaceutical delivery route worldwide, and the choice of excipients fundamentally determines the manufacturability, stability, and performance of the final product. Direct compression (DC) has become the preferred tableting strategy owing to its simplicity, cost-effectiveness, and avoidance of moisture- and heat-sensitive processing steps [1]. However, DC imposes stringent requirements on excipient flowability, compressibility, and blend uniformity that individual raw materials—such as crystalline α-lactose monohydrate or microcrystalline cellulose (MCC)—frequently fail to meet on their own [2,3].

Co-processed excipients (CPEs) address these limitations by combining two or more functional components through particle engineering techniques—including spray drying, co-agglomeration, roller drying, and wet granulation—to produce composite particles with synergistic properties that exceed those of simple physical mixtures [4,5]. The resulting improvements in flowability, compactibility, and dilution potential have been attributed to the intimate sub-particle integration of complementary functionalities: for instance, lactose provides compactibility and flavor masking, while MCC contributes deformability and disintegration [6]. Rojas et al. have demonstrated that co-processing generates enhanced DC functionality compared to physical blending at equivalent compositions [7].

The commercial landscape of lactose-based CPE has expanded considerably, with products from Meggle GmbH (Wasserburg am Inn, Germany) and BASF (Ludwigshafen am Rhein, Germany) representing diverse compositional and manufacturing approaches. Dominik et al. compared four of these products using classical flow and compression testing [8], while Haware et al. characterized MicroceLac 100 using a design-of-mixtures approach [9]. However, these studies relied exclusively on classical methods—compressibility index (CI), Hausner factor, angle of repose, and flow time—which are known to provide only a partial picture of powder behavior.

The limitations of classical pharmacopoeial methods have been extensively documented. Shah et al. demonstrated the poor discriminating power of individual flow tests for pharmaceutical powders [10], while Krantz et al. showed that static and dynamic testing methods probe fundamentally different aspects of powder mechanics [11]. Lindberg et al. compared five flow measurement techniques and found substantial disagreement between methods for cohesive powders [12]. These findings underscore the need for multi-parameter characterization approaches.

The FT4 Powder Rheometer^®^ (Freeman Technology, Tewkesbury, UK) provides precisely such a multi-faceted platform. By its patented rotating blade methodology, the FT4 quantifies dynamic flow resistance (Basic Flowability Energy, BFE), temporal stability (Stability Index, SI), sensitivity to flow rate changes (Flow Rate Index, FRI), and interparticulate cohesion (Specific Energy, SE) in a single instrument [13,14]. Complementary modules enable consolidated tapping, aeration, compressibility, permeability, shear cell, and wall friction measurements, providing over 20 independent flow descriptors per sample. The dynamics of the FT4 blade interaction have been characterized by Hare et al. using high-speed imaging and DEM simulation [15]; correcting numerical errors in that work does not affect the qualitative characterization cited here. Nan and Ghadiri systematically investigated the effects of air flow and particle shape on FT4 measurements [16,17]. Bharadwaj et al. used discrete element method (DEM) simulations to establish the mechanistic relationship between particle properties and FT4 flow energy [18].

Van Snick et al. published a landmark multivariate database of 55 raw materials with over 100 descriptors, demonstrating the utility of combining FT4 data with classical and particle-level measurements for in silico process design [19]. Their subsequent work extended this framework into a multivariate formulation and process development platform [20]. However, their datasets focus predominantly on single-component excipients and APIs, with limited representation of commercially available CPE. Navaneethan et al. used the FT4 to assess lubrication efficiency in pharmaceutical particulate systems [21], and Majerová et al. investigated the effect of colloidal silica on FT4-measured rheological properties of common excipients [22]. Ono and Yonemochi evaluated ibuprofen powder properties using the FT4 in the context of surface modification [23].

The present study addresses the existing knowledge gap by providing the first systematic, side-by-side characterization of fifteen lactose-based excipients—spanning co-processed, spray-dried, spray-agglomerated, and specialty products—across the complete FT4 measurement suite and all relevant Ph. Eur. flow characterization methods. The primary objectives were (1) to establish comprehensive multi-dimensional rheological fingerprints for each material; (2) to quantify the additional information provided by FT4 parameters beyond classical pharmacopoeial methods; (3) to construct a correlation framework linking particle-level attributes to both traditional and advanced flow descriptors; (4) to investigate within-family effects of particle size distribution on flow behavior (Tablettose 70/80/100, FlowLac 90/100); and (5) to evaluate the relationship between measurement variability (standard deviation) and material cohesiveness. The resulting dataset and correlation framework can serve as a decision support tool for rational excipient selection in DC formulation development.

## 2. Materials and Methods

### 2.1. Materials

Fifteen excipient samples representing five processing categories were investigated. Table 1 lists all materials with their declared compositions, manufacturing methods, typical particle morphology, and manufacturers. The materials were categorized as (i) multi-component co-processed excipients (Cellactose 80, MicroceLac 100, Compaction Blend M, CombiLac, StarLac, RetaLac, MicrocelacPlus); (ii) roller-dried/milled anhydrous lactose (DuraLac); (iii) wet granulation product (Ludipress); (iv) spray-agglomerated single-component lactose (Tablettose 70, 80, 100); and (v) spray-dried products (Kollitab, FlowLac 90, FlowLac 100). All materials were used as received from their respective manufacturers without further processing.

The manufacturing method fundamentally determines particle morphology and, consequently, flow behavior. Five distinct manufacturing categories are represented in this study: (a) Co-processing (CP) involves milling, mixing, and sieving of two or more components, producing irregularly shaped particles with moderate flow properties that depend strongly on composition and particle size distribution. Seven materials in this study (Cellactose 80, MicroceLac 100, Compaction Blend M, CombiLac, StarLac, RetaLac, MicroceLacPlus) fall into this category, though they span a wide range of compositions and particle morphologies. (b) Roller drying with subsequent milling (RC), represented solely by DuraLac H, produces dense, angular crystalline particles of anhydrous β-lactose through evaporation of a lactose solution on heated rollers followed by milling and sieving to the desired particle size distribution. (c) Wet granulation (WG), represented by Ludipress, builds granule particles through fluid-bed or mixer granulation with a binder solution, resulting in irregularly shaped granules with moderate sphericity. (d) Spray agglomeration (SPA), used for the Tablettose grades (70, 80, 100), fluidizes a crystalline α-lactose monohydrate powder and sprays water to build up agglomerate granules. The resulting particles have irregular shapes with lower sphericity than spray-dried products, but the agglomeration process creates a bimodal internal structure combining the good compactibility of fine lactose with the flowability of coarser particles. (e) Spray drying (SPD), used for Kollitab, FlowLac 90, and FlowLac 100, atomizes a solution or suspension into a hot air stream. Surface tension during drying produces near-spherical particles (sphericity close to 1.0), which is the primary reason for the consistently superior flow properties of spray-dried products [24,25,26]. Materials in categories (a) through (d) produce non-spherical particles with moderate to poor flow, while category (e) produces spherical particles with consistently good to excellent flow. This systematic ordering (CP → RC → WG → SPA → SPD) is maintained throughout all figures to facilitate visual identification of manufacturing-related trends.

An important functional distinction exists between ready-to-use pre-blended mixtures and single-component excipients. Kollitab DC 87 L and Compaction Blend M are marketed as ready-to-use direct compression bases that already contain an integrated lubricant (1% sodium stearyl fumarate in Kollitab; vegetable magnesium stearate in Compaction Blend M) and, in the case of Compaction Blend M, a disintegrant component [7,27]. These formulations are designed to be mixed directly with the active pharmaceutical ingredient without requiring additional excipient blending steps. The remaining thirteen materials are individual excipients serving specific functions—as fillers, binders, disintegrants, or matrix formers—and must be combined with additional components before tableting. This distinction is critical for interpreting flow data: the ready-to-use mixtures are optimized for overall processability, which explains why Kollitab achieves the best flow properties in the dataset, whereas single excipients like RetaLac or DuraLac are designed to provide specific functionality (sustained release or unique compaction behavior, respectively) even at the cost of poorer flow [4,27,28].

### 2.2. Bulk and Tapped Density (Ph. Eur. 2.9.34)

Bulk density (BD) and tapped density (TD) were determined according to Ph. Eur. 2.9.34 [29] using a 250 mL graduated cylinder (2 mL graduation). Sample masses of 100 g were used for all materials except RetaLac (50 g, due to low bulk density exceeding cylinder volume at 100 g). Materials were gently introduced via a funnel, and the unsettled apparent volume V_0_ was recorded. Tapping was performed in sequences of 10, 500, and 1250 strokes using an Erweka SVM 102 (2008) tapping device according to USP method 2, with a dropping height of 3 mm (250 ± 15 taps/min; tap height 3 ± 0.2 mm). If V_500_ − V_1250_ > 2 mL, additional 1250-stroke cycles were performed until the volume change between consecutive measurements was ≤2 mL. The compressibility index (CI) was calculated as

CI = 100 × (V_0_ − V_e_)/V_0_ [%] (1)

Due to limited sample availability, the CI determination was performed as a single measurement (*n* = 1) per material. However, as discussed in Section 3.6, the measured CI values show excellent correlation with the FT4-derived conditioned bulk density (CBD, r = 0.97 with BD) and tapped density parameters (BDtap50), providing internal validation of the single-measurement approach [24].

### 2.3. Flow Time (Ph. Eur. 2.9.16)

Flow time was measured according to Ph. Eur. 2.9.16 [29] using a standardized stainless steel funnel with the following geometry: upper diameter 125 mm, orifice diameter 12 mm, cone angle 60°, stem length 125 ± 10 mm, material thickness 1.25 mm, and a 45–angled lower orifice. The funnel orifice was blocked with a flat card, and 100 g of material (or 75 g for MCC-rich materials where 100 g exceeded funnel capacity) was introduced. Upon removal of the card, timing was started simultaneously and stopped when the last material exited the funnel. If the powder did not flow, the flow time was recorded as infinite (∞). Each determination was performed in triplicate (*n* = 3), and results are reported as mean ± standard deviation.

### 2.4. Angle of Repose (Ph. Eur. 2.9.36)

The angle of repose was determined according to the principles of Ph. Eur. 2.9.36, using a simplified laboratory-developed method validated against the pharmacopoeial requirements. A funnel (orifice diameter 18 mm) was fixed at a height of 21 mm above millimeter paper. The sample mass was 75 g for all materials (50 g for RetaLac). After blocking the funnel orifice with a card, the powder was gently introduced. Upon card removal, the powder discharged and formed a cone on the millimeter paper. The cone diameter was measured in four cardinal directions (N, S, E, W) and averaged to obtain the mean radius. Each determination was performed in quintuplicate (*n* = 5), providing a more robust statistical basis than the standard triplicate approach. The angle of repose (α) was calculated as

α = arctan(h/(r − r_0_)) (2)

where h = 21 mm (funnel height above surface), r = mean measured cone radius, and r_0_ = 9 mm (funnel orifice radius). Flow behavior was classified according to the scale defined in Ph. Eur. 2.9.36.

### 2.5. Particle Size and Shape Analysis

Particle size distributions were measured by dynamic image analysis (*n* = 1) using the Retsch/Jenoptik Camsizer system (Retsch Technology GmbH, Haan, Germany). This instrument captures two-dimensional projections of individual particles during free-fall and derives volume-weighted size percentiles (d10, d50, d90) based on the minimum chord length xmin, which represents the shortest distance between two parallel tangent lines on the particle projection and is particularly suitable for non-spherical particles. A minimum of 100,000 particles was measured per sample to ensure statistical representativeness, with the span value as a distribution breadth descriptor, the volume-weighted sphericity (SPHT3), and the specific surface area per volume (Sv, 1/mm). The Sauter mean diameter (d32) was calculated as

d32 = 6/Sv(3)

Importantly, the Camsizer operates with dry dispersion and does not apply shear forces sufficient to break apart loosely adhered agglomerates. As a consequence, the measured d10, d50, and d90 values are expected to be systematically larger than those reported by manufacturers, who typically use laser diffraction with wet dispersion and/or sonication that disrupts agglomerates. The Camsizer-measured values therefore represent the effective particle size under conditions more closely resembling actual powder processing, where agglomerates may persist during blending, die filling, and compression [25,26].

### 2.6. FT4 Powder Rheometer Measurements

All FT4 measurements were performed using the FT4 Powder Rheometer^®^ (Freeman Technology, Tewkesbury, UK) with 50 mm diameter borosilicate glass vessels (85 mL for bulk/shear properties and dynamic flow; 260 mL for aeration tests) and a 48 mm blade. The FT4 Powder Rheometer is a universal powder testing instrument that measures flow properties by forcing a precision blade along a helical path through a powder sample contained in a cylindrical vessel. The instrument measures the axial force and torque during both downward (compressive) and upward (shearing) traversals. The total energy consumed during each traverse is calculated from the integral of the combined force and torque resistance over the distance traveled. Each test module was performed in triplicate (*n* = 3) with fresh, conditioned samples. The instrument conditioning procedure (a standardized sequence of downward and upward blade traverses at −48° blade angle and 100 mm/s tip speed) was applied before each test to establish a reproducible low-stress packing state, eliminating operator-dependent packing effects. The 50 mm vessel was selected in accordance with Freeman Technology recommendations for pharmaceutical free-flowing to moderately cohesive excipients with particle sizes < 500 µm, which is the size range covered by all fifteen materials in this study; this vessel size offers an optimal balance between sample requirement (approximately 80 g) and signal quality and allows direct comparability with the majority of published FT4 datasets on pharmaceutical powders [13,19,30]. Although the FT4 instrument also offers a 25 mm bore diameter vessel, the 50 mm vessel has been established as the de facto standard for pharmaceutical excipient characterization; therefore, all measurements in this study were performed exclusively with this vessel size, and inter-vessel comparisons were not within the scope of this work [13].

#### 2.6.1. Stability and Variable Flow Rate (SVFR) Test

The Stability and Variable Flow Rate (SVFR) test is the most fundamental measurement program of the FT4 Powder Rheometer. It consists of two phases: (1) a stability phase comprising seven conditioning and test cycles at a constant blade tip speed of 100 mm/s, and (2) a variable flow rate phase where the blade tip speed is reduced sequentially to 70, 40, and 10 mm/s. The following parameters are derived:

BFE = Flow Energy at Test 7 (100 mm/s) [mJ](4)

The Basic Flowability Energy (BFE) represents the total energy consumed during the seventh downward blade traverse through the conditioned powder bed. It is measured under confined, relatively high-stress conditions where the powder is forced against the base of the vessel. BFE is not a direct measure of flowability but rather reflects the combined effects of interparticulate friction, cohesion, mechanical interlocking, and bulk density [30]. High BFE values can arise from either high interparticulate friction or high bulk density with dense mechanical interlocking.

SI = FE_7_/FE_1_ (Stability Index) (5)

The Stability Index (SI) quantifies the change in flow energy between the first and seventh test cycles. SI values near 1.0 indicate temporal stability of the powder under repeated testing; values > 1.0 suggest progressive compaction or triboelectric charging; values < 1.0 indicate attrition or de-agglomeration during testing.

FRI = FE_10_ mm/s/FE_100_ mm/s (Flow Rate Index) (6)

The Flow Rate Index (FRI) quantifies the material’s sensitivity to changes in blade tip speed. It is calculated as the ratio of flow energy at 10 mm/s to that at 100 mm/s. Values > 1.0 indicate cohesive behavior (higher resistance at lower speeds, as slower blade movement allows cohesive bonds to form), while values < 1.0 suggest free-flowing characteristics where reduced inertial effects dominate.

SE = Upward traverse energy/sample mass [mJ/g](7)

The Specific Energy (SE) is measured during the upward blade traverse and is particularly sensitive to interparticulate cohesion because the upward movement lifts particles against gravity without the confining pressure of the downward stroke. Unlike BFE, SE is independent of bulk density effects and therefore provides a more direct indication of cohesive forces [31]. The Conditioned Bulk Density (CBD, g/mL) is recorded after the conditioning cycle as the baseline bulk density for all subsequent measurements.

#### 2.6.2. Consolidation, Aeration, Compressibility, and Permeability Tests

The consolidation test applied a sequence of 50 taps to derive CEtap50 (tapped flow energy, mJ), CItapped (consolidation index), and BDtap50 (tapped bulk density, g/mL). The tapping was performed manually by hand, as no suitable attachment for an automated tapping device was available for the FT4 instrument at the time of measurement. While this method is less standardized than automated tapping, the resulting BDtap50 values showed good agreement with the tapped densities obtained from the Ph. Eur. 2.9.34 method, providing confidence in the measurement approach. To ensure reproducibility of the manual tapping, a standardized protocol was followed: the FT4 vessel was lifted to a height of approximately 14 mm and allowed to drop under gravity onto a folded laboratory towel, repeating this 50 times per tapping sequence at a consistent rhythm. Different operators performed the tapping throughout the study; however, the fixed drop height and uniform surface ensure negligible operator-to-operator variability. The 14 mm drop height corresponds to USP Method 1 (Erweka SVM 102) used for Ph. Eur. 2.9.34 measurements. The reproducibility of the manual procedure was confirmed by the low triplicate standard deviations of BDtap50 (RSD < 3% for all materials except DuraLac) and by the good agreement between FT4-derived BDtap50 and pharmacopoeial-tapped density (r = 0.95 across all fifteen materials). The systematic component of any manual tapping error therefore propagates equally across the dataset and does not affect between-material comparisons. Nevertheless, the authors acknowledge manual tapping as a methodological limitation, and a mechanized tapping attachment should be used in future work.

Aeration testing measured flow energy at increasing air velocities (0, 2, 4, 6, 8, 10 mm/s) to derive the Aeration Ratio (AR_10_ = BFE/AE_10_) and the Normalized Aeration Sensitivity (NAS, s/mm). According to the FT4 documentation [30], materials can be classified by their aeration behavior: AR ≈ 1 indicates that the powder is not sensitive to aeration (usually very cohesive powders or those containing high levels of binder); 2 < AR < 20 represents average sensitivity to aeration (most powders fall within this range); and AR >> 20 indicates very high sensitivity to aeration, where the powder likely becomes fluidized (typical for pigments (ZnO, TiO_2_, iron oxides, other inorganic and organic color pigments), powder coatings, and powders with very low cohesive strength).

AR_10_ = BFE/AE_10_ (8)

NAS = (BFE − AE_10_)/(BFE × v_10_) [s/mm] (9)

Compressibility was measured as the percentage volume reduction in the powder bed at applied normal stresses of 0.5, 1, 2, 4, 6, 8, 10, 12, and 15 kPa. CPS_15_ (compressibility at 15 kPa) serves as the primary compressibility descriptor. Permeability was quantified as the pressure drop (Δp) across the powder bed under the same normal stress range, with Δp_15_ (at 15 kPa) as the primary descriptor.

#### 2.6.3. Shear Cell Testing

Rotational shear cell measurements (*n* = 3) were performed to construct Mohr–Coulomb yield loci and derive cohesion (kPa), unconfined yield strength (UYS, kPa), major principal stress (MPS, kPa), flow function coefficient (ffc = MPS/UYS), and the angle of internal friction (AIF, °). The selection of the consolidation normal stress (σ_n_) for each material required a preliminary screening procedure: each powder was first tested at multiple normal stresses (typically 2, 3, 6, 9, and 15 kPa) in single measurements. The yield locus data were evaluated for linearity, as the Mohr–Coulomb analysis requires a linear relationship between shear stress and normal stress at the pre-shear and shear points. The normal stress at which the yield locus exhibited the most linear behavior and the lowest scatter was selected for the definitive triplicate determination. This approach ensures that each material is tested under conditions that produce reliable and reproducible yield loci, rather than applying a single normal stress to all materials regardless of their consolidation behavior. The powder must be in a mechanically stable state to obtain meaningful yield loci; insufficient consolidation leads to nonlinear, curved yield loci that violate the Mohr–Coulomb linearity assumption. It should be noted that MPS, UYS, and cohesion are all stress-dependent parameters that change with the applied normal stress, which is why material-specific normal stress selection is essential for obtaining reliable measurements with minimal scatter.

The resulting normal stress assignments ranged from 2 kPa (Compaction Blend M, Tablettose 70—both free-flowing, low-cohesion materials requiring minimal consolidation) to 15 kPa (Ludipress, Tablettose 100, MicrocelacPlus—materials requiring higher consolidation for stable yield loci). Intermediate values included 3 kPa (CombiLac), 6 kPa (Kollitab, RetaLac), and 9 kPa (Cellactose 80, MicroceLac 100, DuraLac, FlowLac 90/100, StarLac, Tablettose 80). The ffc was classified according to Jenike’s scale [32]: ffc < 1 (not flowing), 1–2 (very cohesive), 2–4 (cohesive), 4–10 (easy-flowing), >10 (free-flowing).

#### 2.6.4. Wall Friction Testing

In the wall friction test, the shear stress is measured against a defined metal surface rather than against the powder itself. A wall friction head with defined surface roughness is placed on the pre-consolidated powder bed and rotated under decreasing normal stresses. Three stainless steel wall friction heads with surface roughness of Ra = 0.05 µm (electropolished, mirror-like finish), Ra = 0.28 µm (medium), and Ra = 1.2 µm (relatively coarse) were used, covering the range of surface finishes commonly encountered in pharmaceutical manufacturing equipment. The electropolished surface (Ra < 0.05 µm) is produced by selective electrochemical material removal, achieving a surface quality beyond typical pharmaceutical requirements but providing valuable insights into adhesive wall friction behavior. The normal stress for wall friction testing was matched to the shear cell normal stress selected for each material, ensuring consistent consolidation conditions across both measurements. This coupling is important because wall friction depends not only on surface roughness but also on the powder’s consolidation state.

### 2.7. Statistical Analysis

Due to limited sample availability and/or the labor-intensive nature of certain single-measurement methods, the compressibility index (Ph. Eur. 2.9.34) and particle size/shape analysis were performed as single determinations (*n* = 1). Although *n* = 1 is not ideal from a statistical standpoint and should be considered a methodological limitation of this study, the validity of the individual CI and particle size values is supported by two internal cross-checks: (i) the single-measurement CI values correlate strongly with the triplicate-measured FT4 parameters CBD (r = 0.97) and BDtap50 (r = 0.93), providing indirect replication; and (ii) the Camsizer d50 and SPAN values are themselves statistically robust because each measurement is based on >100,000 individual particles, which provides excellent intra-sample reproducibility even though inter-batch reproducibility was not assessed. All FT4 measurements, flow time (*n* = 3), and angle of repose (*n* = 5) were performed in replicate as described in the respective subsections. Future studies should ideally include the triplicate determination of all pharmacopoeial parameters.

Pearson correlation coefficients (r) were computed pairwise across all 53 measured and derived parameters for the fifteen excipients, producing a 53 × 53 correlation matrix. Correlation strength was classified as strong (|r| > 0.7), moderate (0.5 < |r| ≤ 0.7), or weak (|r| ≤ 0.5). Standard deviations of triplicate FT4 measurements were included as separate parameters in the correlation matrix to investigate the relationship between measurement variability and material properties. All measurements are reported as mean ± standard deviation.

## 3. Results and Discussion

### 3.1. Bulk and Tapped Density, Compressibility Index

**Figure 1:**
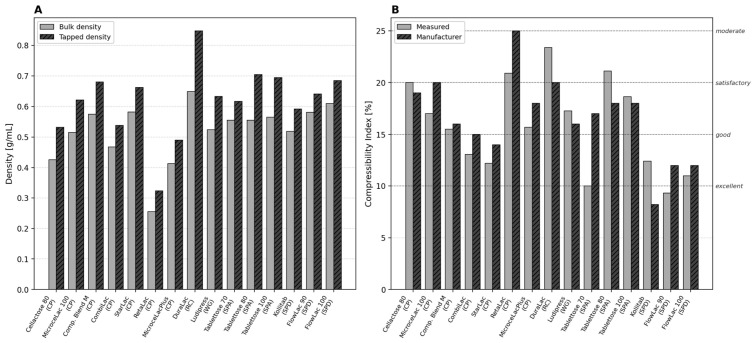
Physical density-related descriptors of the fifteen lactose-based excipients. (**A**) Bulk density (BD) and tapped density (TD) measured according to Ph. Eur. 2.9.34 (single determination, *n* = 1). (**B**) Compressibility index (CI): experimentally measured values (*n* = 1) compared with manufacturer-reported data; classification thresholds according to Ph. Eur. 2.9.36 are indicated: excellent (<10%), good (11–15%), satisfactory (16–20%), moderate (>21%). Materials are ordered by manufacturing method throughout all figures. Gray shades and hatching distinguish the six manufacturing method groups (see legend).

Figure 1A presents the bulk density and tapped density values for all fifteen excipients, ordered by manufacturing method (co-processing, roller compaction, wet granulation, spray agglomeration, spray drying).

Bulk densities spanned from 0.255 g/mL (RetaLac) to 0.650 g/mL (DuraLac), reflecting the structural contrasts between porous HPMC-containing particles and dense anhydrous β-lactose crystals. Tapped densities ranged from 0.323 to 0.848 g/mL along the same axis. The compressibility index, derived from these measurements, classified the materials into four groups regarding flow properties according to Ph. Eur. 2.9.36 (Figure 1B).

The measured CI values generally agreed well with manufacturer data (mean absolute deviation: 2.4 percentage points), with notable exceptions: Tablettose 70 (measured 10.0% vs. manufacturer 17%), and Compaction Blend M (measured 15.5% vs. 16%). The discrepancy for Tablettose 70 likely reflects batch-to-batch variation or differences in the tapping procedure (SVM 102 vs. manufacturer method).

### 3.2. Flow Time and Angle of Repose

**Figure 2:**
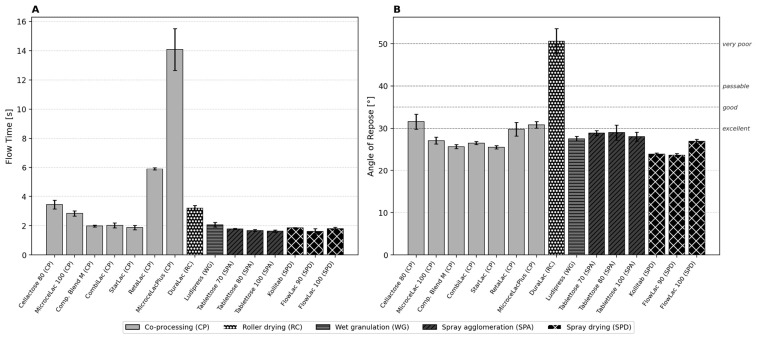
Classical pharmacopoeial flow descriptors. (**A**) Flow time according to Ph. Eur. 2.9.16, measured in triplicate (*n* = 3) with standard deviation error bars; 100 g sample mass (75 g for MCC-rich materials). (**B**) Angle of repose (*n* = 5, mean ± SD) compared with manufacturer-reported values; classification thresholds according to Ph. Eur. 2.9.36 are indicated.

Flow time measurements revealed a wide performance range spanning nearly an order of magnitude (Figure 2A). MicrocelacPlus exhibited by far the longest flow time (14.09 ± 1.43 s), followed by RetaLac (5.90 ± 0.09 s). Both materials contain high proportions of fine or fibrous particles (65% MCC and 50% HPMC K4M, respectively), which create interparticulate bridges in the funnel. FlowLac 90 achieved the shortest flow time (1.62 ± 0.19 s), closely followed by Tablettose 100 (1.63 ± 0.07 s) and Tablettose 80 (1.67 ± 0.06 s). The Tablettose grades showed remarkably low standard deviations, reflecting the consistent flow behavior of spray-agglomerated particles.

The angle of repose data (Figure 2B) generally corroborates the CI-based classification, with DuraLac exhibiting a conspicuously high value (50.6°, classified as very poor—the only material in this category). FlowLac 90 (23.7°) and Kollitab (23.9°) show the lowest angles, classified as excellent. An interesting discrepancy emerges for the Tablettose grades: despite their low CI values and short flow times, they exhibit relatively high repose angles (28.0–28.9°), suggesting that the static pile formed after funnel discharge captures different aspects of particle interaction than the dynamic flow or tapping measurements. This is consistent with the observation of Tharanon et al. that pharmacopoeial flow methods often provide inconsistent rankings [33].

Comparison with manufacturer-reported angles of repose (Figure 2B) reveals generally good agreement for the spray-dried and spray-agglomerated products (mean absolute deviation: 2.8°), while several co-processed products show larger discrepancies. The measured values for the SPD group (Kollitab: 23.9° vs. manufacturer 26.6°; FlowLac 90: 23.7° vs. 27°; FlowLac 100: 26.7° vs. 28°) are systematically lower than manufacturer specifications, likely reflecting differences in the measurement methodology. For the CP group, Cellactose 80 (31.6° vs. 34°) and RetaLac (29.6° vs. 36°) also show lower measured values, whereas DuraLac (RC, 50.6° vs. 42°) is the only material where our measurement exceeds the manufacturer value—consistent with the known sensitivity of DuraLac’s angular β-lactose crystals to funnel geometry and measurement conditions.

### 3.3. Particle Size and Shape Characterization

**Figure 3:**
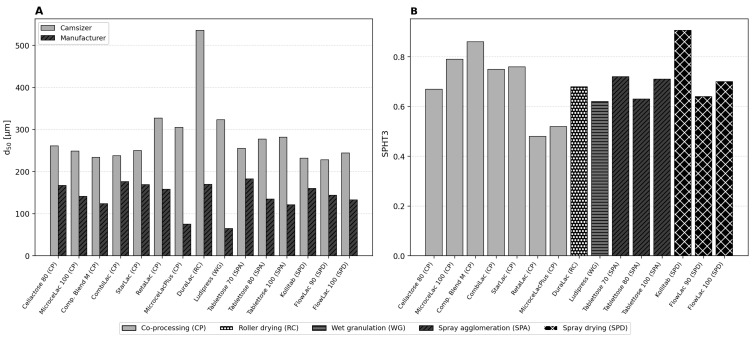
Particle size and shape descriptors from dynamic image analysis (Camsizer, *n* = 1). (**A**) Median particle size d50 compared with manufacturer-reported values; Camsizer values systematically exceed manufacturer values due to agglomerate preservation during dry dispersion. (**B**) Volume-weighted particle sphericity SPHT3 for all fifteen excipients; higher values indicate more spherical particles.

A systematic discrepancy was observed between Camsizer-measured and manufacturer-reported particle sizes (Figure 3A). In all fifteen cases, the volume-weighted Camsizer d50 exceeded the manufacturer value, with ratios ranging from 1.45 (Kollitab: 232 vs. 160 µm) to 4.07 (MicrocelacPlus: 305 vs. 75 µm). The largest deviations occurred for materials known to form strong agglomerates: MicrocelacPlus (65% MCC) and DuraLac (d50: 536 vs. 170 µm, ratio 3.15). Since volume-weighting emphasizes larger particles disproportionately, the presence of agglomerates shifts the d50 upward. This pattern is consistent with the hypothesis that the Camsizer’s dry dispersion preserves loosely adhered agglomerates that are disrupted by wet dispersion and ultrasonic de-agglomeration used in laser diffraction [34].

A systematic discrepancy is observed between Camsizer-measured and manufacturer-reported particle sizes (Figure 3A). In all fifteen cases, the volume-weighted Camsizer d50 exceeds the manufacturer value. Since volume-weighting emphasizes larger particles disproportionately (a single particle of twice the diameter contributes eight times the volume), the presence of agglomerates or coarse particles shifts the d50 upward, with ratios ranging from 1.45 (Kollitab: 232 vs. 160 µm) to 4.07 (MicrocelacPlus: 305 vs. 75 µm). The largest deviations occur for materials known to form strong agglomerates: MicrocelacPlus (65% MCC) and DuraLac (d50: 536 vs. 170 µm, ratio 3.15). From a practical standpoint, the Camsizer values may better represent the effective particle size during actual powder processing [34].

Sphericity (Figure 3B) ranged from 0.480 (RetaLac) to 0.907 (Kollitab). The spray-dried products (Kollitab, Compaction Blend M, FlowLac 90) achieved higher sphericity than agglomerated or milled materials, confirming that the spray-drying process inherently produces more regular particle shapes. The strong correlation between SPHT3 and flow behavior parameters supports the long-established role of particle shape as a primary determinant of powder flowability [35].

### 3.4. Stability and Variable Flow Rate (SVFR) Flow Energy Profiles

**Figure 4:**
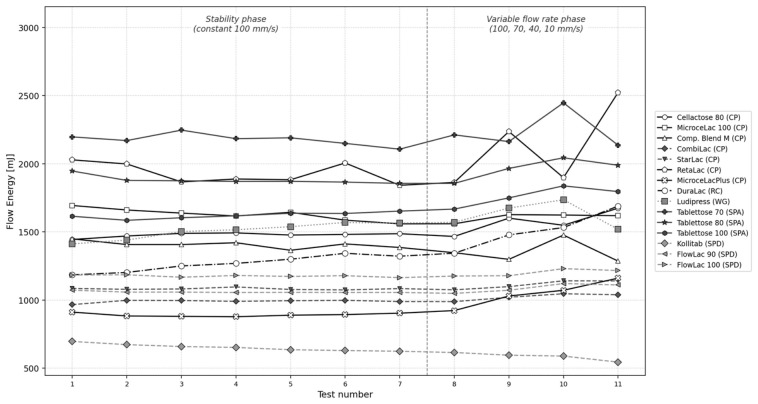
Complete Stability and Variable Flow Rate (SVFR) flow energy profiles for all fifteen excipients (*n* = 3, mean values shown). Tests 1–7 (stability phase) at constant 100 mm/s blade tip speed; Tests 8–11 (variable flow rate phase) at 100, 70, 40, and 10 mm/s, respectively. Error bars are omitted for clarity; BFE standard deviations are shown in Figure 5A. The vertical dashed line separates the stability and variable flow rate phases. Gray shades and line styles distinguish the six manufacturing method groups (see legend).

The complete SVFR flow energy profiles (Figure 4) provide a comprehensive view of the dynamic flow behavior of all fifteen excipients across both the stability phase (Tests 1–7 at 100 mm/s) and the variable flow rate phase (Tests 8–11 at decreasing blade tip speeds). Several distinct behavioral patterns emerge when materials are grouped by the manufacturing method.

The co-processed excipients (CPs) span a wide range of flow energies (approximately 1000–1900 mJ), reflecting the diversity of compositions and particle morphologies within this group. RetaLac shows notably elevated and variable flow energies due to its fibrous HPMC particle network, while CombiLac and StarLac occupy the lower end of the CP range, consistent with their higher sphericity values. Compaction Blend M, despite containing magnesium stearate as an integrated lubricant, shows intermediate flow energies, suggesting that the lubricant reduces interparticulate friction but does not fully compensate for the irregular particle shape.

The spray-agglomerated Tablettose grades (SPA) consistently occupy the highest flow energy range (approximately 1600–2200 mJ), with Tablettose 70 showing the highest BFE in the entire dataset. During the variable flow rate phase (Tests 8–11), the SPA materials show moderate flow energy increases at reduced blade speeds, indicating some cohesive tendency that manifests only under low-shear conditions. The high BFE of spray-agglomerated products is mechanistically attributable to their dense agglomerate structure: the rough, interlocking particle surfaces create high mechanical resistance to blade penetration, even though these materials are not cohesive in the shear cell sense.

In contrast, the spray-dried products (SPD) consistently occupy the lowest flow energy range. Kollitab shows by far the lowest flow energies across all test conditions (approximately 550–700 mJ), reflecting its near-perfect sphericity (SPHT3 = 0.907), narrow particle size distribution (SPAN = 0.338), and integrated SSF lubricant. The FlowLac grades show intermediate SPD behavior (approximately 1000–1200 mJ), higher than Kollitab but still substantially below the CP and SPA groups. Notably, Kollitab exhibits a slight downward trend during the stability phase (SI = 0.90), suggesting minor attrition of the spray-dried surface layer during repeated blade traversals.

The wet granulation product Ludipress (WG) and the roller-compacted DuraLac (RC) occupy intermediate positions. DuraLac shows the highest flow energy variability between replicate measurements, consistent with its angular, irregular crystalline particle morphology and high cohesion.

### 3.5. FT4 Dynamic Flow Properties: Basic Flowability Energy, Specific Energy, Stability Index, and Flow Rate Index

**Figure 5:**
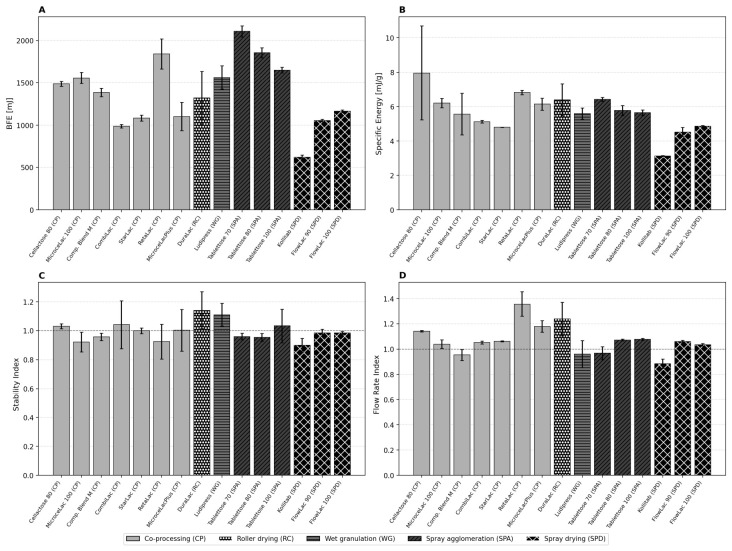
FT4 dynamic flow properties derived from the SVFR test (*n* = 3, mean ± SD). (**A**) Basic Flowability Energy (BFE, test 7 at 100 mm/s). (**B**) Specific Energy (SE) from the upward blade traverse. (**C**) Stability Index (SI); the dashed line at SI = 1.0 represents ideal stability. (**D**) Flow Rate Index (FRI); the dashed line at FRI = 1.0 represents rate-insensitivity.

The BFE (Figure 5A) varied over a fourfold range, from 624 ± 26 mJ (Kollitab) to 2107 ± 64 mJ (Tablettose 70). Crucially, BFE is not a univocal measure of “flowability” but rather reflects the total mechanical energy consumed during forced blade displacement through a conditioned powder bed. High BFE values can arise from either high interparticulate friction (as in RetaLac, BFE = 1842 ± 178 mJ) or high bulk density with dense mechanical interlocking (as in Tablettose 70, BFE = 2107 ± 64 mJ, despite good classical flow indicators). This distinction is critical: Tablettose 70 has high BFE because its densely packed agglomerates create strong mechanical resistance to blade penetration, whereas RetaLac has high BFE due to the fibrous HPMC particles’ entanglement and high interparticulate friction.

The BFE standard deviation itself provides valuable information. DuraLac shows the highest absolute BFE variability (314.6 mJ), followed by RetaLac (177.7 mJ) and MicrocelacPlus (167.5 mJ)—all materials with high cohesion. In contrast, free-flowing materials like FlowLac 90 (15.2 mJ), FlowLac 100 (16.8 mJ), and CombiLac (19.3 mJ) show remarkably low BFE variability. This observation is quantified in the correlation analysis (Section 3.7), where BFE standard deviation correlates strongly with cohesion (r = 0.73) and with the angle of repose (r = 0.84). The implication is significant: measurement reproducibility itself is a material property that tracks cohesion.

Specific Energy (Figure 5B) ranges from 3.14 ± 0.04 mJ/g (Kollitab, SPD) to 7.96 ± 2.72 mJ/g (Cellactose 80, CP). The very low SE of Kollitab reflects its near-perfect sphericity (SPHT3 = 0.907) and integrated sodium stearyl fumarate lubricant, which acts as both a lubricant and an anti-adherent. The high SE of Cellactose 80 is consistent with its cellulose component creating interparticulate bridges, but the exceptionally high standard deviation (2.72 mJ/g, 34% relative) suggests that this material’s SE is highly sensitive to the conditioning state. Again, the spray-dried products consistently show the lowest SE values, while co-processed and roller-compacted products show the highest values.

The Stability Index (Figure 5C) is close to unity for most materials (0.90–1.14), confirming effective conditioning. DuraLac (SI = 1.14) and Ludipress (SI = 1.11) show progressive flow energy increases over repeated tests, potentially indicating mechanical interlocking or triboelectric charging. Kollitab (SI = 0.90) shows the strongest negative deviation, suggesting slight attrition of its spray-dried particles during testing. The FRI (Figure 5D) shows more wide variation: RetaLac exhibits the highest FRI (1.357), indicating that its flow energy increases by 36% when the blade speed decreases from 100 to 10 mm/s—a hallmark of strong cohesive behavior. Kollitab (FRI = 0.885) and Compaction Blend M (FRI = 0.954) show FRI < 1.0, consistent with free-flowing behavior.

### 3.6. FT4 Consolidation, Aeration, Compressibility, and Permeability

The comparison between BFE/SE and shear cell methods reveals important complementarities. BFE and ffc showed no significant linear correlation (|r| < 0.3), because BFE is dominated by bed density while ffc is dominated by cohesion. However, SE and cohesion showed a moderate positive correlation (r = 0.56), confirming that both parameters capture aspects of interparticulate adhesion. The most striking complementarity is illustrated by Tablettose 70: it has the highest BFE in the dataset (2107 mJ) yet a respectable ffc of 13.9 and very low cohesion (0.072 kPa). This apparent contradiction resolves when recognizing that BFE includes a substantial density contribution—Tablettose 70’s dense agglomerate structure creates high mechanical resistance to blade displacement without generating shear failure.

**Figure 6:**
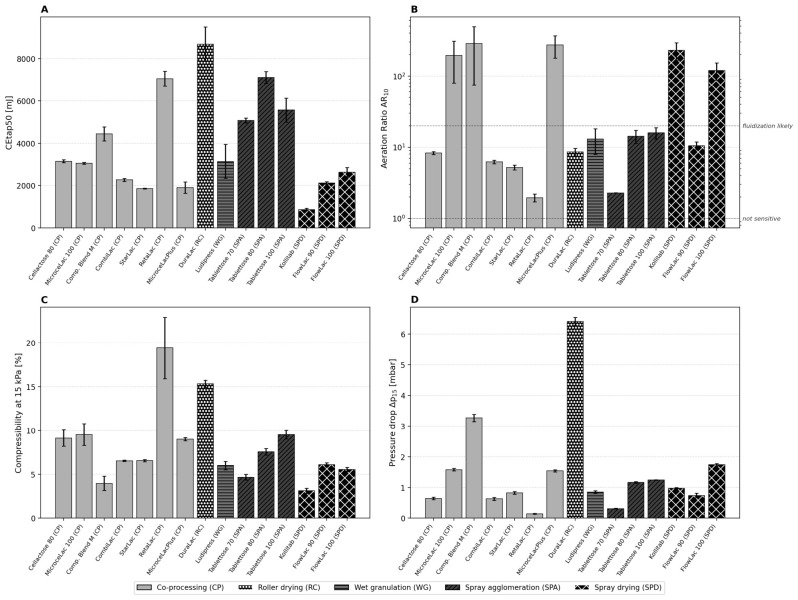
FT4 consolidation, aeration, compressibility, and permeability parameters (*n* = 3, mean ± SD). (**A**) Consolidation energy CEtap50 after 50 taps. (**B**) Aeration Ratio at 10 mm/s (AR_10_) on logarithmic scale; classification thresholds AR ≈ 1 (not sensitive) and AR >> 20 (fluidization likely) are indicated. (**C**) Compressibility at 15 kPa (CPS_15_). (**D**) Pressure drop at 15 kPa (Δp_15_).

The consolidation energy CEtap50 (Figure 6A) shows wide variation: Kollitab requires only 869 ± 54 mJ, while DuraLac demands 8678 ± 807 mJ—a tenfold range. The Tablettose grades show a clear CEtap50 progression, Tablettose 70 (5076 mJ) < Tablettose 100 (5567 mJ) < Tablettose 80 (7112 mJ), which correlates with their SPAN values (0.605 < 0.711 < 1.213) rather than their d50 values.

Aeration behavior (Figure 6B) discriminates sharply between the fifteen excipients. Three distinct groups emerge according to the classification introduced in Section 2.6.2 (AR ≈ 1 = not sensitive; 2 < AR < 20 = average sensitivity; AR >> 20 = fluidization likely): (i) highly sensitive materials that achieve near-complete fluidization at 10 mm/s air velocity (AR_10_ >> 20): Compaction Blend M (283.7), MicrocelacPlus (271.8), Kollitab (228.7), MicroceLac 100 (193.7), FlowLac 100 (120.2); (ii) moderately sensitive materials (2 < AR_10_ < 20): Tablettose 80 (14.3), Ludipress (13.0), FlowLac 90 (10.5), DuraLac (8.6), Cellactose 80 (8.3), CombiLac (6.2), StarLac (5.2); and (iii) aeration-resistant materials (AR_10_ ≈ 1–2): Tablettose 70 (2.3) and RetaLac (1.9). RetaLac’s very low AR_10_ indicates that its fibrous HPMC network physically resists air penetration.

Compressibility profiles (Figure 6C) show characteristic nonlinear increases with applied normal stress. RetaLac reaches the highest CPS_15_ (19.4 ± 3.5%), followed by DuraLac (15.3 ± 0.4%), while Kollitab (3.1 ± 0.3%) and Compaction Blend M (4.0 ± 0.8%) show the lowest compressibility. The RetaLac curve exhibits the steepest initial slope (5.5% at 0.5 kPa), confirming the high deformability of HPMC-containing particles. Notably, RetaLac’s CPS standard deviation (3.5%) is by far the highest, again demonstrating that cohesive materials produce more variable measurements.

Permeability (Figure 6D) varies over a 45-fold range. DuraLac (6.42 ± 0.13 mbar) and Compaction Blend M (3.27 ± 0.12 mbar) form the least permeable beds. RetaLac (0.14 ± 0.02 mbar) maintains the highest permeability despite its high compressibility, due to the open, fibrous network structure that preserves connected air channels even under compression.

The permeability–density interaction pattern warrants further discussion. High-density materials do not universally produce low-permeability beds. RetaLac, despite having the lowest bulk density (0.255 g/mL), showed the lowest pressure drop (0.14 ± 0.02 mbar), while DuraLac, with the highest density (0.650 g/mL), showed the highest (6.42 ± 0.13 mbar). This apparent paradox resolves when considering pore morphology: RetaLac’s fibrous HPMC network creates large, interconnected channels that remain open even under compression, whereas DuraLac’s dense, angular crystals pack tightly, creating tortuous, constricted air pathways. This distinction is critical for high-speed tableting: materials with high pressure drop (DuraLac, Compaction Blend M) require slower turret speeds or vacuum-assisted die filling to achieve consistent tablet weight, while materials with low pressure drop (RetaLac, FlowLac 90) can accommodate higher production speeds [36].

### 3.7. Shear Cell and Wall Friction Analysis

**Figure 7:**
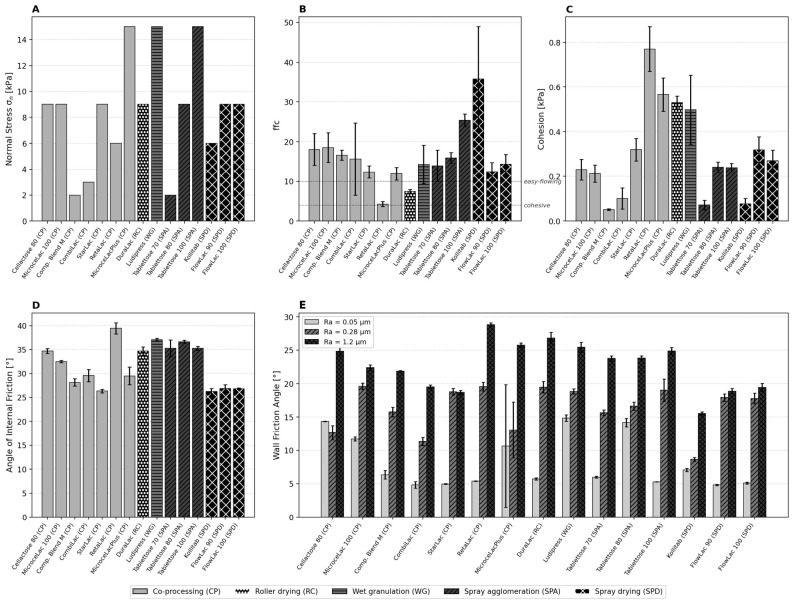
Shear cell and wall friction parameters (*n* = 3, mean ± SD). (**A**) Material-specific normal stress σ_n selected for each material based on yield locus linearity screening. (**B**) Flow function coefficient ffc; classification thresholds according to Jenike [32]. (**C**) Shear cell cohesion. (**D**) Angle of internal friction (AIF). (**E**) Wall friction angle at three surface roughness (Ra = 0.05, 0.28, and 1.2 µm).

The material-specific normal stress selection (Figure 7A) itself provides valuable classification information. Free-flowing materials with low cohesion (Compaction Blend M, Tablettose 70) required only 2 kPa for stable yield loci, while cohesive or dense materials (Ludipress, Tablettose 100, MicrocelacPlus) required 15 kPa. This pattern arises because cohesive powders need sufficient consolidation to form a mechanically stable shear zone; at low normal stresses, the yield locus becomes nonlinear due to incomplete inter-particle contact formation.

The flow function coefficient ffc (Figure 7B) classifies Kollitab (SPD) as the best-flowing material (ffc = 35.8 ± 13.1) and RetaLac (CP) as the only cohesive material (ffc = 4.3 ± 0.6). DuraLac (RC, ffc = 7.4 ± 0.5) is the only pure lactose classified below the free-flowing threshold, attributable to its angular, crystalline particle morphology. Cohesion (Figure 7C) ranges from 0.051 kPa (Compaction Blend M, CP) to 0.770 kPa (RetaLac, CP). The angle of internal friction (Figure 7D) shows that the spray-agglomerated Tablettose grades (35.3–36.6°) have systematically higher AIF than the spray-dried FlowLac grades (26.8–26.9°), suggesting that the interlocking surface roughness of agglomerates creates higher internal friction than the smoother surfaces of spray-dried particles.

Wall friction (Figure 7E) shows a strong and consistent dependence on surface roughness. Kollitab consistently achieves the lowest WFA across all roughnesses (7.0/8.6/15.6°), attributable to its spherical particles and sodium stearyl fumarate as an integrated lubricant and anti-adherent.

The wall friction data at three roughnesses revealed an important practical insight for equipment design. For most materials, the wall friction angle increased monotonically with surface roughness (0.05 → 0.28 → 1.2 µm). However, Cellactose 80, Tablettose 80, and Ludipress showed anomalously high WFA at the smoothest surface (Ra = 0.05 µm: 14.4°, 14.2°, and 14.8°, respectively), approaching their WFA values at Ra = 0.28 µm. This suggests that for certain materials, the electropolished surface creates stronger adhesive contact—possibly through increased van der Waals interactions due to larger true contact area—than a slightly rougher surface where asperities reduce the effective contact zone. This finding is relevant for the design of pharmaceutical equipment surfaces: electropolishing does not universally reduce wall friction and may even increase it for specific powder–surface combinations.

### 3.8. Correlation Analysis and Key Relationships

The Pearson correlation matrix across all 53 parameters reveals several families of strong relationships. The most important correlations, ordered from highest to lowest absolute correlation coefficient, are summarized in Table 2.

**Figure 8:**
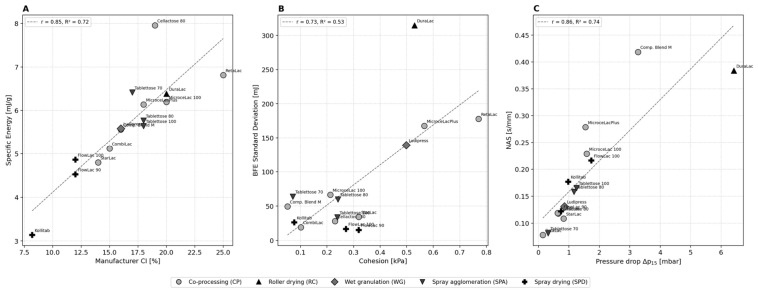
Key bivariate correlations identified from the 53 × 53 Pearson correlation matrix. (**A**) Specific Energy (SE) vs. manufacturer-reported compressibility index (CI) [r = 0.85, R^2^ = 0.72]. (**B**) Basic Flowability Energy standard deviation vs. shear cell cohesion [r = 0.73, R^2^ = 0.53]; cohesive materials produce less reproducible dynamic flow measurements. (**C**) Normalized Aeration Sensitivity (NAS) vs. pressure drop at 15 kPa (Δp_15_) [r = 0.86, R^2^ = 0.74]; both parameters probe the pore structure of the powder bed. Marker shape and shade distinguish manufacturing method groups.

The strongest bivariate correlation in the dataset links manufacturer-reported compressibility index (CI) to Specific Energy (Figure 8A, r  =  0.85, R^2^  =  0.72). This relationship is mechanistically coherent: CI reflects the degree of volume reduction upon tapping, which depends on particle shape, surface roughness, and cohesion—the same properties that determine interparticulate friction during the upward blade traverse measured by SE. The correlation confirms that SE can serve as a rapid, instrument-based surrogate for the classical CI screening test, providing equivalent ranking information with greater sensitivity to differences within the “good-to-fair” CI range where most CPE reside.

The correlation between BFE standard deviation and cohesion (Figure 8B, r = 0.73) provides a novel insight: the reproducibility of dynamic flow energy measurements is itself a material property that correlates with cohesion. This finding has practical implications for quality control: if triplicate BFE measurements show high variability, this alone suggests cohesive behavior even before calculating ffc or cohesion from shear cell data.

The NAS–Δp_15_ correlation (Figure 8C, r = 0.86) connects two independently measured properties that probe the same underlying physical attribute: the interconnected pore network of the packed bed. Materials with high NAS (slow decrease in flow energy with increasing air velocity) also show high pressure drops (low permeability). This redundancy enables method reduction: where testing time or material is limited, either NAS or permeability can be measured alone [36].

## 4. Discussion

### 4.1. Within-Family Comparisons: Tablettose and FlowLac Grades

The Tablettose grades (SPA, spray agglomeration; 70, 80, 100) and FlowLac grades (SPD, spray drying; 90, 100) provide unique within-family comparisons where the base material (α-lactose monohydrate) is chemically identical but processed by different manufacturing methods. Within each family, the target particle size varies—at Meggle, larger numbers indicate smaller particle sizes. This allows the investigation of particle size distribution effects independently of composition.

For the Tablettose grades, the measured d50 values (Camsizer) are 255, 277, and 282 µm for T70, T80, and T100, respectively—a relatively narrow range. However, the SPAN values diverge considerably: 0.605 (T70), 1.213 (T80), and 0.711 (T100). This SPAN variation correlates strongly with CI: T70 (SPAN = 0.605, CI = 10.0%), T100 (SPAN = 0.711, CI = 18.6%), T80 (SPAN = 1.213, CI = 21.1%). The relationship is mechanistically clear: broader distributions allow fine particles to fill interparticulate voids upon tapping, producing greater volume reduction and higher CI [37,38].

The FlowLac comparison (90 vs. 100) shows that despite similar manufacturing process and base material, FlowLac 90 outperforms FlowLac 100 on CI (9.3% vs. 11.0%), SE (4.52 vs. 4.86 mJ/g), and angle of repose (23.7° vs. 26.9°). However, FlowLac 100 shows considerably higher aeration sensitivity (AR_10_ = 120.2 vs. 10.5 for FlowLac 90), indicating that FlowLac 100 would be preferred for air-assisted die filling, while FlowLac 90 would be superior for gravity-fed processes.

### 4.2. The Role of Particle Shape: Sphericity as a Master Variable

Particle sphericity (SPHT3) emerges as the single most consistent predictor of flow behavior across both classical and FT4 methods. Materials with SPHT3 > 0.85 (Kollitab: 0.907, Compaction Blend M: 0.861) consistently achieve the best flow performance: lowest BFE, lowest SE, highest ffc, and shortest flow times. Conversely, materials with low sphericity (RetaLac: 0.480, MicroceLacPlus: 0.519) rank among the poorest performers on virtually all flow indicators. The mechanistic explanation follows directly from contact mechanics: spherical particles have minimal contact area per unit volume, reducing van der Waals adhesion and mechanical interlocking. Additionally, spherical particles pack more efficiently under gravity, creating homogeneous powder beds that respond reproducibly to both gravitational and forced-flow testing. This finding supports the observation by Wolf (verbal communication) that the manufacturing method is the primary determinant of flow behavior through its effect on particle shape: only spray drying (method e) consistently produces near-spherical particles, while all other methods (a through d) produce irregular shapes with inherently poorer flow [24,25,35,39].

### 4.3. Measurement Variability as a Cohesion Indicator

An underappreciated aspect of powder characterization is the information contained in measurement variability. In this study, the standard deviations of triplicate FT4 measurements were included as explicit parameters in the correlation matrix, revealing systematic relationships between variability and material cohesiveness. The BFE standard deviation correlated strongly with cohesion (r = 0.73), angle of repose (r = 0.84), and SPAN (r = 0.61). This pattern indicates that cohesive materials with broad particle size distributions produce fundamentally less reproducible dynamic flow measurements, even under carefully controlled FT4 conditioning.

The mechanistic explanation is straightforward: in cohesive powders, the conditioning procedure cannot fully eliminate history-dependent packing variations. Cohesive interparticulate forces (van der Waals, electrostatic, capillary) create metastable packing states that vary between replicate measurements despite identical conditioning protocols. In contrast, free-flowing materials like FlowLac 90 (BFE SD = 15.2 mJ, 1.4% relative) rapidly achieve equilibrium packing under conditioning, yielding highly reproducible results.

The observation that BFE standard deviation and other FT4 variability metrics correlate with material cohesion has not been extensively discussed in the literature. Our data suggest that for cohesive materials, the conditioning procedure cannot fully eliminate history-dependent packing variations, leading to inherently less reproducible measurements. This effect propagates to the shear cell as well: the ffc standard deviation for Kollitab (13.12, Figure 7B) is paradoxically the highest in the dataset despite its high mean ffc (35.8), because at very low-cohesion levels, small absolute variations in cohesion produce large relative variations in ffc.

These findings have practical implications for quality control: high measurement variability in routine FT4 testing should be interpreted not merely as a sign of poor analytical precision but as a material property indicator. If triplicate BFE measurements yield a coefficient of variation exceeding approximately 10%, the material is likely to exhibit cohesive behavior and may require special handling during manufacturing.

### 4.4. Basic Flowability Energy and Specific Energy vs. Shear Cell Methods: Complementary or Redundant?

To specifically address the question whether classical pharmacopoeial flow descriptors (CI, Hausner ratio) agree with the shear cell-derived ffc—which is routinely used in production for powder flow assessment [1,10]—we computed pairwise Pearson correlations. The correlation between measured CI (Ph. Eur. 2.9.34) and ffc is only moderate (r = −0.52, *p* < 0.05): materials classified as “good” or “excellent” by CI are typically also classified as “free-flowing” or “easy-flowing” by ffc, but the rank order within each category differs substantially. A striking example is Tablettose 70 (CI = 10.0%, “excellent” by CI) which has ffc = 13.9 (free-flowing) vs. FlowLac 90 (CI = 9.3%, “excellent” by CI) which has ffc = 12.7 (free-flowing)—essentially indistinguishable by CI but differing by almost 10% in ffc. The Hausner ratio correlates with ffc slightly better (r = −0.61), but similar category overlaps occur. Crucially, three materials misclassify between the two methods: DuraLac is “fair/passable” by CI (23.4%) but only just-easy-flowing by ffc (7.4)—the shear test identifies it as more cohesive than CI suggests—while MicroceLacPlus is “fair” by CI (15.7%) but free-flowing by ffc (22.9) due to its high aeration sensitivity. The general finding is that CI/Hausner and ffc agree on the qualitative “good flow” vs. “cohesive” dichotomy but disagree on the fine-grained ranking within the good flow group, which is often the decision-relevant region in DC formulation. This confirms the long-standing recommendation that CI and Hausner ratio are useful pre-screening tools but should not be used as the sole flow criterion for DC excipient selection [10,12,33].

These findings reinforce the multi-parameter approach advocated by Freeman and Price [30]: no single test or parameter can fully characterize powder flow behavior, because different processing operations impose different mechanical stress states on the powder. Hopper discharge is best predicted by ffc and WFA; die filling depends on aeration behavior and permeability; blending performance relates to BFE and SI; and feeding consistency depends on SE and FRI.

A central question in powder rheological characterization is whether the dynamic FT4 parameters (BFE, SE, FRI) and the quasi-static shear cell parameters (cohesion, ffc, AIF) provide complementary or redundant information. Our correlation analysis clearly supports the complementarity hypothesis. BFE and ffc show no significant linear correlation (|r| < 0.3), confirming that these parameters measure fundamentally different mechanical phenomena: BFE probes forced flow under blade displacement (relevant to mixing, blending, and feeding), while ffc probes yield behavior under normal stress (relevant to hopper discharge and flow initiation). A material can have high BFE yet excellent ffc (Tablettose 70: BFE = 2107 mJ, Figure 5A; ffc = 13.9, Figure 7B) if its flow energy arises from packing density rather than cohesion. Conversely, RetaLac shows both high BFE (1842 mJ, Figure 5A) and poor ffc (4.3, Figure 7B), where cohesion is the common driver.

SE shows a moderate correlation with cohesion (r = 0.56), which is expected because both parameters are sensitive to interparticulate adhesion. The FRI shows the strongest correlation with cohesion (r = 0.79, Table 2), suggesting that rate sensitivity in dynamic flow is the closest FT4 analog to shear cell cohesion.

### 4.5. Detailed Analysis of Individual Material Performance

The co-processed excipients (CP) in this study deserve particular attention because they span the widest range of flow properties despite sharing the same general manufacturing category. This heterogeneity reflects fundamental differences in composition, particle morphology, and intended function. Cellactose 80 (75% α-lactose monohydrate + 25% powdered cellulose; diluent/binder combination) and MicroceLac 100 (75% α-lactose monohydrate + 25% microcrystalline cellulose) combine a lactose filler with a cellulose-based binder/disintegrant, producing angular, irregular particles with moderate cohesion (CI = 20.0% and 17.1%, respectively). MicroceLacPlus reverses this ratio (35% lactose + 65% MCC), creating significantly more fibrous particles (SPHT3 = 0.519) with the longest flow time in the dataset (14.09 ± 1.43 s) but very high aeration sensitivity (AR_10_ = 271.8), which would be advantageous in pneumatic conveying or air-assisted die filling systems. CombiLac (70% lactose + 20% MCC + 10% maize starch) and StarLac (85% lactose + 15% maize starch) represent simpler binary and ternary systems with intermediate flow properties. RetaLac (50% α-lactose monohydrate + 50% HPMC K4M) is unique as the only sustained-release matrix former in the dataset [28]; its deliberately high polymer content creates the fibrous particle network responsible for gel formation upon hydration but also produces the poorest flow properties (ffc = 4.3, the only cohesive material). Compaction Blend M (α-lactose monohydrate + MCC + aluminum oxide + magnesium stearate) achieves notably low cohesion (0.051 kPa, lowest in dataset) through its integrated lubricant, demonstrating that even irregular CP particles can achieve good flow when surface modification reduces interparticulate adhesion [40,41].

DuraLac (RC) occupied a unique position as the only anhydrous β-lactose in the dataset. Its high bulk density (0.650 g/mL, highest measured), very high consolidation energy (CEtap50 = 8678 ± 807 mJ), and lowest ffc among pure lactoses (7.4) reflect the angular, dense crystalline morphology of roller-dried β-lactose crystals. The pressure drop at 15 kPa (6.42 mbar, highest in dataset by a factor of 2.0) confirmed that DuraLac forms dense, low-permeability beds, which is directly problematic for high-speed rotary tablet presses where air must escape rapidly from the die cavity during filling.

MicrocelacPlus, a newer product with a reversed composition ratio compared to MicroceLac 100 (65% MCC + 35% LMH instead of 75% LMH + 25% MCC), showed interesting intermediate behavior. Its flow time was considerably longer (14.09 ± 1.43 s vs. 2.84 ± 0.18 s for MicroceLac 100), its BFE lower (1102 vs. 1559 mJ), and its aeration sensitivity much higher (AR_10_ = 271.8 vs. 193.7). The higher MCC content creates more fibrous, elongated particles (SPHT3 = 0.519 vs. 0.786), explaining the poorer gravity-driven flow.

Kollitab (SPD) emerges as the best-performing material across nearly all flow indicators: lowest BFE (624 ± 26 mJ, Figure 5A), lowest SE (3.14 ± 0.04 mJ/g, Figure 5B), highest ffc (35.8, Figure 7B), lowest wall friction angles (Figure 7E), lowest angle of repose (23.9°, Figure 2B), and shortest flow time among CPE products (1.85 s, Figure 2A). This exceptional performance can be attributed to three synergistic factors: (1) the spray-drying process produces entirely spherical particles (SPHT3 = 0.907); (2) the narrow particle size distribution (SPAN = 0.338) minimizes interparticulate bridging; and (3) the ~1% sodium stearyl fumarate acts as an integrated lubricant and anti-adherent, reducing both interparticulate friction and wall friction.

RetaLac (CP) represents the opposite end of the spectrum. Its high HPMC content (50%) creates a fibrous particle network that produces the highest cohesion (0.770 kPa, Figure 7C), the lowest ffc (4.3, Figure 7B), the highest FRI (1.357, Figure 5D), and the highest compressibility (CPS_15_ = 19.4%, Figure 6C). However, RetaLac also shows two unique properties: the highest bed permeability (Δp_15_ = 0.14 mbar, Figure 6D) and the highest compressibility. These properties are functional requirements for its intended application as a sustained-release matrix former.

Each manufacturing method offers distinct advantages that must be weighed against flow performance when selecting excipients for a specific formulation. Co-processing (CP) provides the greatest formulation flexibility: by combining multiple functional components at the particle level, CP products can simultaneously deliver filler, binder, and disintegrant functionality in a single excipient, simplifying the manufacturing process and reducing the number of unit operations [4,7]. However, as demonstrated in this study, CP products show the widest variability in flow properties (BFE range: 993–1842 mJ within the CP group alone), reflecting the inherent trade-off between multifunctionality and flow optimization. Roller compaction (RC), represented by DuraLac, produces dense anhydrous β-lactose crystals with unique brittle fracture behavior under compression, making it valuable for roller compaction/dry granulation applications despite its poor flow characteristics (ffc = 7.4, highest Δp_15_ = 6.42 mbar) [42]. Wet granulation (WG), represented by Ludipress, creates granules with good compactibility through the PVP binder bridges between lactose crystals, providing tablets with acceptable hardness at relatively low compression forces [39]. Spray agglomeration (SPA) combines the compactibility of fine lactose with the flowability of coarser agglomerates (BFE: 1665–2107 mJ), though the rough agglomerate surfaces produce higher internal friction angles (AIF: 35.3–36.6°) than spray-dried products [38]. Spray drying (SPD) consistently achieves the best flow properties through near-spherical particle morphology (SPHT3: 0.786–0.907), but the resulting particles are often mechanically fragile and may exhibit polymorphic instability if the amorphous content is high [8,43]. The choice between these methods thus represents a multi-objective optimization problem where flow performance is only one of several critical quality attributes.

### 4.6. Comprehensive Correlation Framework: Implications for Formulation Development

The second cluster links cohesion-related parameters (SE, FRI, cohesion, CPS_15_, WFA, BFE SD) with inter-correlations of 0.56–0.85. This cluster captures the spectrum of interparticulate adhesion effects and identifies materials likely to cause manufacturing problems.

The third cluster links pore structure parameters (NAS, Δp, AR_10_, permeability) with inter-correlations of 0.72–0.86. This cluster is critical for high-speed tableting where die filling depends on rapid air displacement. Materials with high Δp and low AR values (Tablettose 70, RetaLac, DuraLac) would require slower turret speeds or vacuum-assisted filling. Conversely, materials with low Δp and high AR (Compaction Blend M, MicrocelacPlus, Kollitab) can accommodate high turret speeds because air escapes easily from the die cavity.

The framework established in this study extends the approach of Van Snick et al. [19,20] to commercially available CPE systems and provides manufacturer-specific benchmarks. The correlation coefficients reported here can be used for in silico prediction of unmeasured parameters: for example, if a formulation scientist has SE data from a routine SVFR test, the manufacturer CI can be estimated from the linear regression (R^2^ = 0.72).

Practical decision rules for DC excipient selection emerging from this dataset can be summarized as follows: (1) For a process-robust blend with tight weight uniformity at high turret speeds, choose materials with ffc > 15, AR_10_ > 20, and Δp_15_ < 3 mbar; Kollitab, Compaction Blend M, and the Tablettose grades meet all three criteria. (2) If the main formulation constraint is low API loading and high dilution potential, spray-dried fillers with SPHT3 > 0.85 (Kollitab, FlowLac 90) are the first choice, as their narrow SPAN (<0.5) minimizes risk of segregation. (3) If the formulation requires a disintegrant-binder co-excipient for immediate-release tablets, Ludipress or CombiLac are the preferred options because they combine an acceptable flow profile (ffc > 10) with established functional performance. (4) For modified-release matrix tablets, RetaLac remains the only viable option in the dataset despite its poor flow (ffc = 4.3, FRI = 1.357) and will generally require the addition of a glidant (0.5–1% colloidal silicon dioxide) and a flow-improving lactose (e.g., blend with FlowLac 90 at 30–50%). (5) If die fill consistency is the critical concern on a high-speed rotary press, materials with AR_10_ < 5 (DuraLac, Tablettose 70) should be avoided unless a force-feeder or vacuum-assisted fill is available. These rules are necessarily heuristic and based on a finite dataset of fifteen materials, but they illustrate how the correlation framework can be operationalised.

The correlation analysis reveals three major parameter clusters. The first cluster links classical density-based parameters (BD, TD, CI, CBD, BDtap50) with r values ranging from 0.93 to 0.98. The practical implication is that FT4-derived CBD can substitute for classical BD when material is limited. The second cluster links cohesion-related parameters (SE, FRI, cohesion, CPS_15_, WFA, BFE standard deviation) with inter-correlations of 0.56–0.85. The third cluster links pore structure parameters (NAS, Δp, AR_10_, permeability) with inter-correlations of 0.72–0.86, which is critical for high-speed tableting where die filling depends on rapid air displacement.

The negative correlation between ffc and FRI (r = −0.68, Table 2) provides a simple screening rule: if FRI > 1.10, the material is likely to have ffc < 15 and may exhibit cohesive behavior during gravity discharge. This correlation enables early-stage excipient screening without the need for shear cell testing. For the fifteen materials in this study, the FRI > 1.10 criterion correctly identifies all materials with ffc < 12 (DuraLac RC, RetaLac CP, MicrocelacPlus CP, Ludipress WG) and correctly excludes all materials with ffc > 15 except Cellactose 80 CP (FRI = 1.14, ffc = 18.0), yielding a sensitivity of 100% and specificity of 91% for detecting problematic flow behavior. As recommended by the reviewer, a systematic multivariate analysis (e.g., principal component analysis and partial least-squares regression) linking the complete 53-parameter flow-property fingerprints to downstream tableting critical quality attributes (tablet hardness, disintegration time, content uniformity) would further consolidate the patterns observed here [44]. Such an analysis is beyond the scope of the present characterization study and is planned as a dedicated follow-up investigation.

### 4.7. Camsizer vs. Manufacturer Particle Size Data: Implications for Process Relevance

It is important to recognize that optimal flow performance is not the universal design goal for all excipients. Single-component excipients are selected for specific functional attributes: RetaLac provides sustained drug release through HPMC gel formation [28], DuraLac offers unique compaction properties through brittle fracture of anhydrous β-lactose [42], and the Tablettose grades provide an established balance of flowability and compactibility for standard direct compression applications [38,39]. The fact that these materials show poorer flow performance than the ready-to-use mixtures is not a deficiency but rather reflects their design purpose: they are intended to be blended with complementary excipients (including flow aids and lubricants) before processing, whereas Kollitab and Compaction Blend M are already optimized for immediate use. The comprehensive flow characterization presented in this study enables formulators to quantify exactly how much flow improvement is needed when incorporating a poorly flowing but functionally essential excipient into a DC formulation, as well as to select the appropriate compensating measures (e.g., addition of 0.5–1% colloidal silicon dioxide, blending with a high-sphericity excipient, or using air-assisted die filling) based on the specific deficiency identified by FT4 testing [22,40].

### 4.8. Synthesis: FT4 Rheometry vs. Classical Pharmacopoeial Methods

As one of the central objectives of this study, we now synthesize the comparative findings between the complete FT4 measurement suite and the three classical European Pharmacopoeia flow descriptors (compressibility index per Ph. Eur. 2.9.34, flow time per Ph. Eur. 2.9.16, and angle of repose per Ph. Eur. 2.9.36). The comparison is organized along three axes: (i) discriminating power, (ii) mechanistic interpretability, and (iii) practical decision support.

(i) Discriminating power. The three classical methods, taken together, classified 13 of the 15 excipients into a single “good-to-satisfactory flow” bracket (CI = 9–18%, angle of repose 23–32°, flow time 1.6–3.5 s), leaving only RetaLac, DuraLac, and MicroceLacPlus as outliers. Within the “good-to-satisfactory” group, classical ranking was essentially random—e.g., FlowLac 90 (CI = 9.3%) and Tablettose 70 (CI = 10.0%) appear functionally equivalent by CI, whereas FT4 shows that Tablettose 70 has a BFE approximately twice as high (2107 vs. 1055 mJ) and an aeration ratio more than 4.5 times lower (2.3 vs. 10.5). FT4 parameters produce a fourfold range in BFE, a ninefold range in ffc, a 45-fold range in permeability, and an approximately 150-fold range in aeration ratio—the raw data span is orders of magnitude greater than for the classical descriptors and allows for material-specific fine discrimination that is invisible to the pharmacopoeial approach.

(ii) Mechanistic interpretability. Classical descriptors are single-number aggregates of multiple underlying mechanisms. The compressibility index, for example, reflects both interparticulate cohesion and packing geometry but cannot separate them; the angle of repose reflects friction, cohesion, and particle shape simultaneously; the flow time reflects all of the above plus funnel geometry interactions. The FT4 parameter set disentangles these contributions: SE and cohesion probe interparticulate adhesion specifically; AIF probes internal friction; SPHT3 (Camsizer) and FRI probe shape and rate sensitivity; AR_10_ and Δp_15_ probe pore structure. This mechanistic resolution enables root-cause analysis when a powder exhibits problematic flow. The classical descriptors, in contrast, only register the symptoms. A concrete example: Compaction Blend M and Kollitab both have CI ≈ 12–16%, but the FT4 dataset reveals that Compaction Blend M’s good flow arises primarily from the integrated magnesium stearate (low cohesion = 0.051 kPa) whereas Kollitab’s good flow arises primarily from near-perfect sphericity (SPHT3 = 0.907). This difference has direct consequences for formulation: adding an extra lubricant to Compaction Blend M yields diminishing returns, whereas Kollitab responds well to further lubricant addition because its low cohesion is not already optimized.

(iii) Practical decision support. Classical methods remain valuable as rapid pre-screening tools and for regulatory compliance (CI and angle of repose are pharmacopoeial, well-standardized, require minimal instrumentation, and are accepted by every regulatory agency worldwide). They are adequate for clear go/no-go decisions between known extremes (e.g., FlowLac 90 vs. RetaLac). However, in the middle range—where most commercial CPE reside and where the majority of formulation decisions are actually made—classical methods lack the resolution to support rational excipient selection. Our dataset shows that the combination of a minimal FT4 suite (SVFR for BFE/SE/FRI, one shear cell measurement at an appropriate normal stress for ffc, and an aeration test for AR_10_) provides the decisive additional discriminating power at the cost of approximately 2–3 h of additional instrument time per material. The correlation framework established in Section 3.8 further enables method reduction: for routine characterization, SE can substitute for CI (r = 0.85), NAS for Δp_15_ (r = 0.86), and BFE standard deviation can serve as an early indicator of cohesion (r = 0.73 with shear cell cohesion). We therefore recommend, as a practical workflow, that classical methods be retained for regulatory documentation but that FT4 data be used as the primary decision variable in DC excipient selection.

In conclusion, classical and FT4 methods are not interchangeable and should not be viewed as competing approaches. They differ fundamentally in mechanical stress regime (quasi-static/gravitational vs. dynamic/forced), sample history (non-conditioned vs. conditioned), and information content (aggregate single numbers vs. mechanism-resolved multi-parameter fingerprints). The classical methods retain their role as the regulatory standard and as a rapid screening layer; FT4 rheometry provides the process-relevant, formulation-actionable information required for modern DC development. The correlations reported in this study allow seamless translation between the two frameworks.

To provide a concise overview that enables at-a-glance comparison across all fifteen excipients and across the six manufacturing method subgroups, Table 3 summarizes the key flow indicators together with qualitative strengths and limitations for each material. This consolidation is intended to serve as a quick reference for formulators during excipient selection and complements the detailed section-by-section analysis given above.

### 4.9. Limitations of the Study

Several limitations should be noted. First, the compressibility index was determined from a single measurement (*n* = 1) per material due to limited sample availability; however, the strong correlations with multiply determined FT4 density parameters mitigate this concern. Second, the shear cell measurements were performed at material-specific normal stresses, which optimizes measurement quality but complicates direct inter-material comparison, as cohesion, UYS, and ffc are known to be stress-dependent [32]. Third, the study was conducted at multiple environmental conditions (different temperatures and relative humidity); powder flow behavior is known to be sensitive to moisture content and humidity even though the powders were measured at a time directly after opening the 1 kg sample bags [45]. Fourth, the consolidation tapping for CEtap50 was performed manually rather than with an automated device, which may introduce additional variability. Future studies should include automated tapping, multiple humidity conditions, and tabletability testing to link the observed flow differences to actual tablet quality attributes.

## 5. Conclusions

This study provides the first comprehensive, side-by-side characterization of fifteen commercially available lactose-based excipients using the complete FT4 Powder Rheometer measurement suite in combination with all relevant European Pharmacopeia flow characterization methods. The key findings are as follows: (1) Classical flow methods (CI, flow time, angle of repose) classified most materials as having good-to-satisfactory flow properties but failed to discriminate between materials with fundamentally different dynamic flow profiles. (2) FT4 testing revealed considerably wider parameter ranges (fourfold in BFE, ninefold in ffc) and identified material-specific behaviors invisible to classical testing. (3) Ordering materials by manufacturing method (co-processing, roller compaction, wet granulation, spray agglomeration, spray drying) reveals clear trends: spray-dried products consistently show the best flow properties due to their spherical particle morphology, while co-processed and roller-compacted products show more variable and generally poorer flow. (4) The BFE standard deviation correlates with cohesion (r = 0.73), establishing measurement variability as a cohesion indicator. (5) Within-family comparisons demonstrate that particle size distribution breadth (SPAN) is more critical than median particle size for predicting consolidation and compressibility behavior. (6) The established correlation framework enables rational excipient selection and method reduction for DC formulation development.

(1) Classical pharmacopoeial methods provide useful initial screening but fail to resolve process-relevant differences between materials classified in the same flow category. FT4 parameters—particularly SE, FRI, AR_10_, and ffc—offer substantially more sensitive discriminating power.

(2) Specific Energy (SE) correlates strongly with manufacturer CI (r = 0.85) and can serve as a more sensitive, instrument-controlled alternative for interparticulate cohesion assessment.

(3) BFE standard deviation correlates with shear cell cohesion (r = 0.73) and the angle of repose (r = 0.84), establishing measurement variability as a novel indicator of material cohesiveness.

(4) The NAS–permeability correlation (r = 0.86) enables method reduction: either parameter can substitute for the other when testing time or material is limited.

(5) Within-family comparisons (Tablettose 70/80/100, FlowLac 90/100) demonstrate that particle size distribution breadth (SPAN) is a more critical determinant of flow behavior than median particle size alone.

(6) Particle sphericity (SPHT3) is the single most consistent predictor of both classical and advanced flow behavior across all fifteen materials. Materials with SPHT3 > 0.85 consistently outperform those with lower sphericity.

The comprehensive dataset and correlation framework presented herein provide a reference database for rational excipient selection in direct compression formulation development and can serve as a benchmark for evaluating novel co-processed excipient candidates.

## Figures and Tables

**Table 1:** Overview of excipient materials, declared compositions, manufacturing methods, typical particle morphology, and manufacturers. CPE = co-processed excipient; MCC = microcrystalline cellulose; HPMC = hydroxypropyl methylcellulose; PVP = polyvinylpyrrolidone; RC = roller compaction; WG = wet granulation; SPA = spray agglomeration; SPD = spray drying.

Excipient	Composition	Manufacturing Method	Typical Particle Shape	Manufacturer
Cellactose^®^ 80	75% α-lactose monohydrate + 25% powdered cellulose	Co-processing (CPE)	Irregular, angular	Meggle GmbH
MicroceLac^®^ 100	75% α-lactose monohydrate + 25% microcrystalline cellulose (MCC)	Co-processing (CPE)	Irregular, angular	Meggle GmbH
Compaction Blend M	α-lactose monohydrate, MCC, aluminum oxide, vegetable magnesium stearate	Co-processing (CPE)	Irregular, angular	Meggle GmbH
CombiLac^®^	70% α-lactose monohydrate + 20% MCC + 10% maize starch	Co-spray drying (CPE)	Irregular to rounded	Meggle GmbH
StarLac^®^	85% α-lactose monohydrate + 15% maize starch	Spray drying (CPE)	Spherical to rounded	Meggle GmbH
RetaLac^®^	50% hydroxypropyl methylcellulose (HPMC K4M) + 50% α-lactose monohydrate	Co-processing (CPE)	Irregular, fibrous	Meggle GmbH
MicrocelacPlus	35% α-lactose monohydrate + 65% MCC	Co-processing (CPE)	Irregular, angular	Meggle GmbH
DuraLac^®^ H	approximately 80% anhydrous β-lactose + 20% α-lactose (anhydrous form)	Roller drying/milling (RC)	Angular, crystalline	Meggle GmbH
Ludipress^®^	93% lactose + 3.5% Kollidon^®^ 30 (polyvinylpyrrolidone, water-soluble binder) + 3.5% Kollidon^®^ CL (crospovidone, cross-linked PVP, water-insoluble superdisintegrant)	Wet granulation (WG, CPE)	Irregular, granular	BASF
Tablettose^®^ 70	α-lactose monohydrate (100%)	Spray agglomeration (SPA)	Irregular, rough agglomerates	Meggle GmbH
Tablettose^®^ 80	α-lactose monohydrate (100%)	Spray agglomeration (SPA)	Irregular, rough agglomerates	Meggle GmbH
Tablettose^®^ 100	α-lactose monohydrate (100%)	Spray agglomeration (SPA)	Irregular, rough agglomerates	Meggle GmbH
Kollitab^®^ DC 87 L	~87% α-lactose monohydrate + ~9% Kollidon^®^ CL-F (crospovidone, cross-linked PVP, water-insoluble superdisintegrant) + ~3% Kollicoat^®^ IR (PVA-PEG graft copolymer, water-soluble binder) + ~1% sodium stearyl fumarate (lubricant and anti-adherent)	Spray drying (SPD, CPE)	Spherical (near-perfect)	BASF
FlowLac^®^ 90	α-lactose monohydrate (100%)	Spray drying (SPD)	Spherical	Meggle GmbH
FlowLac^®^ 100	α-lactose monohydrate (100%)	Spray drying (SPD)	Spherical	Meggle GmbH

**Table 2:** Selected correlations from the Pearson correlation matrix (53 × 53) across all fifteen excipients, ordered by decreasing absolute correlation strength. n.s. = not significant at *p* < 0.05.

Parameter Pair	r	Interpretation
Bulk Density vs. Tapped Density	0.98	Packing rank order conserved
Bulk Density vs. Conditioned Bulk Density (FT4)	0.97	FT4 conditioning preserves density hierarchy
Angle of repose vs. d10 particle size	0.96	Confounded by shape; irregular large particles form steep cones
Normalized Aeration Sensitivity vs. Pressure Drop	0.86	Both probe pore structure of powder bed
Specific Energy vs. Compressibility Index (manufacturer)	0.85	SE captures CI-like cohesion information with greater sensitivity (Figure 8A)
BFE Standard Deviation vs. Angle of repose	0.84	Cohesive materials produce variable dynamic measurements
Cohesion vs. Flow Rate Index	0.79	Rate-sensitive materials have higher shear cohesion
Cohesion vs. Compressibility at 15 kPa	0.78	Compressible powders tend to be more cohesive
Flow Rate Index vs. Compressibility Index (manufacturer)	0.76	Rate sensitivity correlates with compressibility
BFE Standard Deviation vs. Cohesion	0.73	Measurement variability tracks material cohesion (Figure 8B)
Cohesion vs. SPAN	0.71	Broader PSDs produce more cohesive powders
Flow function coefficient vs. Flow Rate Index	−0.68	Free-flowing powders are rate-insensitive
Basic Flowability Energy vs. Flow function coefficient	n.s.	BFE and ffc measure different mechanical phenomena

**Table 3:** Comprehensive summary of flow and rheological performance of all fifteen lactose-based excipients grouped by manufacturing method. Values show the mean (triplicate *n* = 3, except CI and PSD *n* = 1). Units: CI [%], BFE [mJ], SE [mJ/g], AR_10_, Δp_15_ [mbar], SPHT3. For each manufacturing method group a short qualitative comment summarizes distinct advantages and limitations. CI, compressibility index (Ph. Eur. 2.9.34); ffc, flow function coefficient; BFE, Basic Flowability Energy; SE, Specific Energy; AR_10_, Aeration Ratio at 10 mm/s; Δp_15_, pressure drop at 15 kPa; SPHT3, volume-weighted sphericity; AoR, angle of repose.

Excipient	CI (%)	ffc	BFE	SE	AR_10_	Δp_15_	SPHT3	Key Strengths	Key Limitations
CP-mill—Co-processing by milling/mixing/sieving: irregular particles, multi-component fillers									
Cellactose^®^ 80	20.0	18.0	1487	7.96	8.3	0.88	0.67	Good compactibility through 25% cellulose binder; cost-effective diluent/binder combination	High SE and BFE SD; rough particle shape causes higher WFA on 0.05 µm surface
MicroceLac^®^ 100	17.0	18.5	1559	6.20	193.7	0.21	0.79	Combined filler/disintegrant functionality; very high aeration sensitivity ideal for air-assisted die fill	Moderate flow time; irregular MCC-containing particles increase friction
Compaction Blend M	15.5	16.6	1386	5.56	283.7	0.17	0.86	Lowest cohesion in dataset (0.051 kPa) through integrated MgSt; highest AR_10_; complete ready-to-use DC base	MgSt-lubricated—adding extra glidant yields diminishing returns; contains aluminum oxide
RetaLac^®^	20.9	4.3	1842	6.81	1.9	0.14	0.48	Unique sustained-release matrix former; highest permeability (open HPMC fiber network); highest compressibility (19.4%)	Cohesive (only ffc < 5 in dataset); highest FRI (1.357, strong rate sensitivity); fibrous particles cause high WFA
MicroceLacPlus	15.7	22.9	1102	6.14	271.8	0.20	0.52	Very high aeration sensitivity; high MCC content improves compactibility and hardness at low force	Longest flow time (14.09 s) due to fibrous particles; poor gravity-driven die fill
CP-SPD—Co-processing by co-spray-drying: rounded particles, improved flow vs. CP-mill									
CombiLac^®^	13.1	15.6	988	5.12	6.2	0.63	0.75	Balanced CPE with filler (70% lactose) + binder (20% MCC) + disintegrant (10% starch) in one particle	Moderate aeration sensitivity; intermediate flow behavior, not optimal for any single extreme
StarLac^®^	12.2	12.4	1084	4.80	5.2	0.83	0.76	Binary lactose–starch CPE with short flow time; excellent disintegration from maize starch	Starch may absorb moisture during storage; moderate AR_10_ limits high-speed fill
RC—Roller drying/milling: dense, angular anhydrous β-lactose crystals									
DuraLac^®^ H	23.4	7.4	1321	6.38	8.6	6.42	0.68	Unique anhydrous β-lactose with brittle fracture compaction; highest bulk density (0.650 g/mL); suitable for moisture-sensitive APIs	Highest CEtap50 (8678 mJ); highest Δp_15_ (6.42 mbar, 46× RetaLac); highest AoR (50.6°)
WG—Wet granulation: granular particles with binder bridges									
Ludipress^®^	17.3	14.3	1564	5.58	13.0	0.85	0.62	PVP-bridged granules give good tablet hardness at low compression force; built-in disintegrant (3.5% crospovidone)	Broadest SPAN (1.136) of the group causes segregation risk; high SI (1.11) suggests progressive compaction
SPA—Spray agglomeration: fluidised α-lactose monohydrate + water spraying									
Tablettose^®^ 70	10.0	13.9	2107	6.41	2.3	0.32	0.72	Short flow time (1.78 s); excellent CI (10.0%); good flow + compactibility balance	Highest BFE in dataset (dense agglomerates); low AR_10_ limits high-speed die fill
Tablettose^®^ 80	21.1	15.9	1856	5.77	14.3	1.17	0.63	Balanced flow + compactibility; average aeration sensitivity suits most production equipment	Broadest SPAN (1.213) causes elevated CI despite fast flow time
Tablettose^®^ 100	18.6	25.4	1653	5.64	15.9	1.25	0.71	Highest ffc among Tablettose grades; finer particles give better compactibility at equal pressure	Requires 15 kPa normal stress for stable yield locus—handles more like a cohesive powder
SPD—Single-component spray drying: near-spherical particles, narrow SPAN, consistently best flow									
Kollitab^®^ DC 87 L	12.4	35.8	624	3.14	228.7	0.98	0.91	Best overall flow performer (lowest BFE, highest ffc, lowest WFA); ready-to-use DC base	Low BFE may be insufficient in force-fed blenders; slight SI < 1 suggests surface attrition
FlowLac^®^ 90	9.3	12.5	1055	4.52	10.5	0.74	0.64	Excellent CI (9.3%); low AoR (23.7°); high amorphous content gives smoother surfaces and reduced friction	Amorphous content (~40–50%) may recrystallise during storage; lower d50 than FlowLac 100
FlowLac^®^ 100	11.0	12.4	1164	4.86	120.2	1.74	0.70	Very high aeration sensitivity (AR_10_ = 120.2); predominantly crystalline, physically stable during storage	Slightly higher CI and AoR than FlowLac 90; larger d50 reduces compactibility at equal force

## Data Availability

The original contributions presented in this study are included in the article. Further inquiries can be directed to the corresponding author.

## Abbreviations

The following abbreviations are used in this manuscript:

AIF	Angle of Internal Friction
AR	Aeration Ratio
BD	Bulk Density
BFE	Basic Flowability Energy
CBD	Conditioned Bulk Density
CI	Compressibility Index
CP	Co-Processing
CPE	Co-Processed Excipient
CPS	Compressibility
DC	Direct Compression
ffc	Flow Function Coefficient
FRI	Flow Rate Index
HPMC	Hydroxypropyl Methylcellulose
MCC	Microcrystalline Cellulose
MPS	Major Principal Stress
NAS	Normalized Aeration Sensitivity
PVP	Polyvinylpyrrolidone
RC	Roller Compaction
SD	Standard Deviation
SE	Specific Energy
SI	Stability Index
SPA	Spray Agglomeration
SPD	Spray Drying
SSF	Sodium Stearyl Fumarate
SVFR	Stability and Variable Flow Rate
TD	Tapped Density
UYS	Unconfined Yield Strength
WFA	Wall Friction Angle
WG	Wet Granulation
